# Predictors of self-rated oral health in Canadian Indigenous adults

**DOI:** 10.1186/s12903-021-01796-6

**Published:** 2021-09-06

**Authors:** Ahmed Hussain, Sheyla Bravo Jaimes, Alexander M. Crizzle

**Affiliations:** 1 College of Dentistry, University of Saskatchewan, Saskatoon, SK S7N 5E4 Canada; 2 School of Public Health, University of Saskatchewan, 104 Clinic Place, Saskatoon, SK S7N 2Z4 Canada

**Keywords:** Indigenous adults, Oral health, Dental health perceptions

## Abstract

**Objectives:**

The purpose of this study was to: (1) compare oral health indicators between Indigenous adults and the general population and (2) examine the predictors of poor self-rated oral health in the Indigenous population.

**Methods:**

Data from the 2017–2018 cycle of the Canadian Community Health Survey was used and included 943 Indigenous and 20,011 non-Indigenous adults. Independent variables included demographic information, lifestyle behaviours, dental concerns and care utilization, and transportation access. The dependent variable was self-rated oral health. A logistic regression was performed to determine predictors of poor self-rated oral health.

**Results:**

More than half of the Indigenous sample were aged between 35 and 64 years (57.3%); 57.8% were female. Compared to the general population, the Indigenous group were significantly more likely to have no partner, have less post-secondary education, and have an income of less than $40,000. Almost a fifth of the Indigenous sample self-rated their oral health as poor (18.5%) compared to 11.5% in the general population. Indigenous participants reported significantly poorer general health, had poorer oral care practices, and lifestyle behaviours than the general population (all *p* < .001). Indigenous adults having poor self-rated oral health was predicted by poorer general health, being a smoker, male, bleeding gums, persistent pain, feeling uncomfortable eating food, avoiding foods, and not seeking regular dental care.

**Conclusions:**

There are many predictors of poor self-rated oral health, many of which are preventable. Providing culturally adapted oral health care may improve the likelihood of Indigeneous adults visiting the dentist for preventative care.

## Introduction

Oral health is a strong indicator of overall general health and quality of life [1]. Studies have reported relationships between poor oral health with diabetes, arthritis, dementia, and cardiovascular and respiratory diseases [2]. Additionally, poor oral health can restrict activities of daily living and diminish a person’s quality of life due to pain, functional limitations (e.g., eating, talking) or psychosocial discomfort [3]. There are numerous factors that increase the risk of poor oral health such as metabolic systemic diseases, medications, radiation, poor oral hygiene, sugar rich diet, poor access to dental care, tobacco and alcohol consumption, and an individual’s perceptions of oral health [3, 4].

Perceptions of oral health play a key role in determining one’s behaviours towards oral health practices. Based on Bandura’s self-efficacy theory [5], it is possible that those who perceive oral health problems as serious or requiring intervention are more likely to engage in preventative oral health care such as routine dental visits, brushing, and flossing [5]. Conversely, those who do not perceive their oral health to be poor (despite the contrary) may not seek dental services to maintain their oral health. Studies examining the self-rated oral health show that socioeconomic status, education, age, sex, ethnicity/race, and health status can influence oral health behaviours [6, 7]. Negative oral health perceptions are more frequent among individuals with oral conditions (e.g. pain, functional and aesthetic problems) and vulnerable groups such as those with low income, individuals living in rural or remote areas and Indigenous people [7, 8].

Prior studies examining self-rated oral health have focused on the general Canadian population [6, 7], low-income groups [9], pregnant women [10], and older adults. However, there has only been a few studies that have examined the oral health of the Indigenous population. Prior Canadian studies with Indigenous people show that they have poorer oral health practices (less frequency of teeth brushing or regular dental visits), a higher prevalence of dental caries and periodontal diseases, untreated tooth decay compared to the general Canadian population [11–15]. Additionally, a 2020 study by Mehra and colleagues that used data from 2014 found that Indigenous people (aged 12 and older) have poorer dental care use, and those with poorer oral health were 5.8x more likely to make emergency dental visits [16]. While all these studies include self-rated oral health as an independent variable, none has examined the factors that influence self-rated oral health as an outcome variable. Understanding the factors associated with poor self-rated oral health may provide an opportunity to devise tailored interventions needed to improve general oral health in Indigenous populations. This study seeks to (1) compare oral health indicators between Indigenous adults and the general population; and (2) examine the predictors of poor self-rated oral health in the Indigenous population.

## Materials and methods

### Data source

Data was analyzed using the 2017–2018 cycle of the Canadian Community Health Survey (CCHS). The CCHS is a cross-sectional survey conducted by Statistics Canada, that gathers health-related information of Canadians aged 12 years and older living in the 10 provinces and 3 territories. However, residents living on reserves, in long-term care, full-time members of Canadian Forces, children aged 12–17 living in foster care, and those living in Nunavik and Terres-Cries-de-la-Baie-James (Quebec) are excluded. Detailed methodology and sampling characteristics of the CCHS are described elsewhere [17]. The 2017–2018 CCHS is the most recent CCHS data available, released in June 2020. Data of the 2017–2018 CCHS was collected using the CCHS questionnaire designed for computer-assisted interviewing. The average time to complete the survey was 50 min and was performed in person or over the telephone. Approximately 75% of the data was collected by telephone interviews and 25% in person. The response rate for the 2017–2018 CCHS was 60.7% (187,132 individuals were contacted to participate in the survey). This study was exempt from requiring Research Ethics Board approval consistent with performing secondary data analysis on publicly available datasets.

### Inclusion/exclusion criteria

Participants were included if they were Indigenous, aged 18 and older, living in Ontario, have complete data for Indigenous identification and oral health variables. Of all the provinces and territories, only Ontario participants completed questions on oral health. Indigenous participants self-identified as being First Nations with or without Indian Status, Métis or Inuit. Participants with “Indian Status” refers to a legal status providing a range of rights and benefits offered by the federal, provincial or territorial Canadian governments [18]. For comparison purposes, all non-Indigenous participants were also required to be between the ages of 18 and older and living in Ontario. The analysis excluded participants who were younger than 18 years old and those with incomplete (missing) data for any of the oral health variables examined. The final sample included 943 Indigenous and 20,011 non-Indigenous participants. The total number of participants excluded due to missing data was 2944 (164 Indigenous and 2780 non-Indigenous participants).

### Independent variables

Independent variables include demographics (e.g. age, sex, marital status, education), general health, oral health, lifestyle behaviours, and access to transportation. All independent variables included in the study were categorized by Statistics Canada with further collapsing of variables conducted by the research team. For the purpose of this study, age was categorized as 18–34 years, 35–64 years, and 65 years and older. Sex was coded as female or male and marital status as “partner” (married or common law) or “no partner” (single, divorced or widowed). Education was divided into 2 categories: secondary education or less and post-secondary education. Income was coded as “less than $40,000” and “$40,000 or more” Employment status was categorized as employed or unemployed.

Health variables included self reported general and mental health which were coded as “excellent”, “very good”, “good”, “fair” and “poor” in the CCHS. These were collapsed to good (excellent, very good and good) versus poor (fair and poor). Additionally, participants reported chronic conditions using pre-defined diseases based on the CCHS survey. The conditions on the CCHS included arthritis, asthma, diabetes, effects of stroke, heart disease, high blood pressure, mood (e.g. depression, bipolar disorder, mania or dysthymia) or anxiety disorders (e.g. phobia, obsessive-compulsive disorder, panic disorder). The presence of each chronic condition was coded as “yes” or “no”.

Oral health variables included access to types of dental insurance coverage (yes/no), dental visits for routine maintenance (yes/no) and emergency visits (yes/no), and questions on whether it was uncomfortable to eat particular foods, persistent pain, bleeding gums and bad breath, all categorized as “often”, “sometimes”, “rarely”, and “never” on the CCHS. All these variables were then dichotomized into yes (often/sometimes) and no (rarely/never) categories.

Variables on lifestyle behaviours included smoking status and alcohol consumption in the prior year. Smoking status was coded as “smoker” and “non-smoker”. Alcohol consumption was classified as being a “drinker” and “non-drinker.” Access to transportation was coded as driving a car within the last year (yes/no).

### Outcome variable

The primary outcome variable was self-rated oral health. Self-rated oral health was based on the following question: “In general, would you say the health of your mouth is” 1 = excellent, 2 = very good, 3 = good, 4 = fair and 5 = poor. The variable was further collapsed to good (excellent, very good and good) and poor (fair and poor).

### Statistical analysis

As all variables were categorical, frequencies and valid percent were used to describe the sample. Differences between Indigenous and non-Indigenous responses, as well as the association between independent and outcome variables were examined using Pearson chi-square tests. To account for multiple comparisons, *p* values were corrected by using Bonferroni correction (*p* = .0016 for groups comparisons and *p* = .0017 for associations between independent and dependent variables). To determine predictors of self-rated oral health, a binary logistic regression was performed providing the odds ratio (OR), adjusted odds ratio (AOR), and associated confidence intervals (all variables with *p* < .20 were included in the regression model). The Significance level of the final model was *p* < .05. To account for the complex sampling design, bootstrap weights were applied and provided with the data file by Statistics Canada prior to the statistical analysis. All the analyses were conducted using SPSS (version 27).

## Results

### Sample characteristics

The sample included 943 Indigenous and 20,011 non-Indigenous participants. More than 50% of the Indigenous sample were between the ages of 35 and 64 years; 57.8% were female. Slightly over 50% of the Indigenous sample had no partner and approximately one third had secondary education (33.1%). About 40% were unemployed and 36% had incomes of $40,000 or less. Just over one fifth of the Indigenous sample did not have access to transportation (21.2%). Approximately 20 and 13% rated their general and mental health as being poor. Almost one fifth of the Indigenous sample reported having poor self-rated oral health.

As shown in Table 1, compared to non-Indigenous participants, the Indigenous sample was significantly older and less likely to be married, to have post-secondary education and to have income of $40,000 or greater (all *p* < .001). Additionally, Indigenous adults were significantly more likely to rate their general and mental health as poor compared to non-Indigenous participants, and were more likely to report having anxiety, asthma, diabetes and mood disorders. Compared to the general population, there was a significantly greater proportion of Indigenous respondents who smoked and were regular drinkers.

**Table 1:** Comparison of socio-demographic, health and oral health factors between Indigenous and non-Indigenous people

Variables	Indigenous people n = 943	Non-Indigenous people n = 20,011	Significance *p* < .0016
***Socio-demographic factors***			
*Age*			
18–34	288 (30.5)	4420 (22.1)	*p < .001*
35–64	540 (57.3)	10,160 (50.8)	
65+	115 (12.2)	5431 (27.1)	
*Sex*			
Male	398 (42.2)	8903 (44.5)	*p* = .168
Female	545 (57.8)	11,108 (55.5)	
*Marital status*			
Partner (married or common law)	433 (46.2)	11,022 (55.2)	*p < .001*
No partner (divorced, widowed or single)	504 (53.8)	8949 (44.8)	
*Education*			
Secondary graduation or less	302 (33.1)	4685 (24)	*p < .001*
Post-secondary education	611 (66.9)	14,813 (76)	
*Income*			
Less than $40,000	339 (36.1)	4226 (21.1)	*p < .001*
$40,000 or more	599 (63.9)	15,774 (78.9)	
*Employment status*			
Employed	565 (59.9)	11,730 (58.6)	*p* = .436
Unemployed	378 (40.1)	8274 (41.4)	
***Health-related factors***			
*Perceived general health*			
Good	749 (79.6)	17,470 (87.4)	*p < .001*
Poor	192 (20.4)	2523 (12.6)	
*Perceived mental health*			
Good	816 (86.6)	18,234 (91.3)	*p < .001*
Poor	126 (13.4)	1742 (8.7)	
*Anxiety disorder*			
Yes	191 (20.3)	2269 (11.4)	*p < .001*
No	750 (79.7)	17,712 (88.6)	
*Arthritis*			
Yes	251 (26.6)	5280 (26.5)	*p* = .896
No	691 (73.4)	14,680 (73.5)	
*Asthma*			
Yes	131 (14)	1890 (9.5)	*p < .001*
No	808 (86)	18,102 (90.5)	
*Diabetes*			
Yes	118 (12.5)	1735 (8.7)	*p < .001*
No	823 (87.5)	18,263 (91.3)	
*Effects of stroke*			
Yes	19 (2)	267 (1.3)	*p* = .077
No	922 (98)	19,730 (98.7)	
*Heart disease*			
Yes	58 (6.2)	1201 (6)	*p* = .848
No	881 (93.8)	18,734 (94)	
*High blood pressure*			
Yes	216 (23)	4364 (21.9)	*p* = .411
No	723 (77)	15,593 (78.1)	
*Mood disorder*			
Yes	195 (20.8)	2371 (11.9)	*p < .001*
No	744 (79.2)	17,617 (88.1)	
***Oral health***			
*Dental Insurance Coverage*			
Yes	685 (73.1)	13,131 (65.9)	*p < .001*
No	252 (26.9)	6808 (34.1)	
*Employer Insurance*			
Yes	440 (47.1)	11,120 (55.9)	*p < .001*
No	494 (52.9)	8765 (44.1)	
*Non-Insured health benefits*			
Yes	159 (17)	4 (0)	*p < .001*
No	775 (83)	19,881 (100)	
*Social service insurance*			
Yes	90 (9.6)	798 (4)	*p < .001*
No	844 (90.4)	19,087 (96)	
*Dental visit for check-ups*			
Yes	676 (72.1)	15,690 (78.9)	*p < .001*
No	261 (27.9)	4189 (21.1)	
*Dental visit for emergency*			
Yes	161 (17.1)	2281 (11.4)	*p < .001*
No	780 (82.9)	17,696 (88.6)	
*Uncomfortable to eat foods*			
Yes	209 (22.2)	3092 (15.5)	*p < .001*
No	734 (77.8)	16,919 (84.5)	
*Avoid particular foods*			
Yes	165 (17.5)	2184 (10.9)	*p < .001*
No	778 (82.5)	17,827 (89.1)	
*Persistent pain*			
Yes	168 (17.8)	2328 (11.6)	*p < .001*
No	775 (82.2)	17,683 (88.4)	
*Bleeding gums*			
Yes	278 (29.5)	4351 (21.7)	*p < .001*
No	665 (70.5)	15,660 (78.3)	
*Bad breath*			
Yes	189 (20)	2795 (14)	*p < .001*
No	754 (80)	17,216 (86)	
***Lifestyle behaviours***			
*Smoking Status*			
Smoker	316 (33.5)	3692 (18.5)	*p < 0.001*
Non-smoker	627 (66.5)	16,312 (81.5)	
*Alcohol consumption*			
Drinker	762 (81.2)	16,798 (84.2)	*p* = .012
Non-drinker	177 (18.8)	3148 (15.8)	
***Transportation***			
*Driving last year*			
Yes	743 (78.8)	17,784 (88.9)	*p < .001*
No	200 (21.2)	2224 (11.1)	
***Outcome***			
*Self-rated oral health*			
Good	769 (81.5)	17,702 (88.5)	*p < .001*
Poor	174 (18.5)	2309 (11.5)	

*p* values in italic indicate statistical significance

### Oral health

Compared to the general population, a significantly greater proportion of Indigenous adults rated their oral health as poor (18.5% vs. 11.5%). Indigenous adults, compared to the general population, were significantly more likely to report feeling uncomfortable eating foods (22% vs. 15.5%), avoiding particular foods (17.5% vs. 10.9%), having persistent pain in mouth (17.8% vs. 11.6%), bleeding gums (29.5% vs. 21.7%), bad breath (20% vs 14%), and were more likely to make emergency dental care visits (17.1% vs. 11.4%), although they were less likely to make visits to the dentist for regular maintenance (72.1% vs. 78.9%) than the general population.

Indigenous adults were significantly more likely to have dental insurance (primarily through the Non-Insured Health Benefits and social insurance) and less likely to have employer-based insurance than the general population.

### Comparisons of Indigenous adults with good and poor oral health

As shown in Table 2, Indigenous adults who reported poor self-rated oral health were more likely to have a lower income and be unemployed (both *p* ≤ .001). Indigenous adults with poor self-rated oral health were also significantly more likely to rate their general and mental health as being poor compared to those with good self-rated oral health (*p* < .001). Additionally, Indigenous adults reporting a stroke, anxiety, arthritis, and mood disorders were more likely to have poor self-rated oral health (all *p* ≤ .001). Those rating their oral health as poor were significantly more likely to be smokers and have restricted access to transportation (*p* < .001)..

**Table 2:** Comparisons between perceptions of oral health with demographic factors, health, lifestyle behaviours, and oral health practices in Indigenous adults (N = 943)

Variables	Perceived oral health		Significance *p* < .0017
	Good n = 769	Poor n = 174	
***Socio-demographic factors***			
*Age*			
18–34	230 (29.9)	58 (33.3)	*p* = .530
35–64	447 (58.1)	93 (53.4)	
65+	92 (12)	23 (13.2)	
*Sex*			
Male	316 (41.1)	82 (47.1)	*p* = .146
Female	453 (58.9)	92 (52.9)	
*Marital Status*			
Partner	363 (47.5)	70 (40.7)	*p* = .108
No Partner	402 (52.5)	102 (59.3)	
*Education*			
Secondary education or less	234 (31.5)	68 (40)	*p* = .033
Post-secondary education	509 (68.5)	102 (60)	
*Income*			
Less than $40,000	243 (31.8)	96 (55.2)	*p < .001*
$40,000 or more	521 (68.2)	78 (44.8)	
*Employment status*			
Employed	486 (63.2)	79 (45.4)	*p < .001*
Unemployed	283 (36.8)	95 (54.6)	
***Health-related factors***			
*Perceived general health*			
Good	654 (85.3)	95 (54.6)	*p < .001*
Poor	113 (14.7)	79 (45.4)	
*Perceived mental health*			
Good	692 (90.1)	124 (71.3)	*p < .001*
Poor	76 (9.9)	50 (28.7)	
*Anxiety disorder*			
Yes	129 (16.8)	62 (35.8)	*p < .001*
No	639 (83.2)	111 (64.2)	
*Arthritis*			
Yes	188 (24.5)	63 (36.2)	*p= .0015 *
No	580 (75.5)	111 (63.8)	
*Asthma*			
Yes	103 (13.5)	28 (16.1)	*p* = .367
No	662 (86.5)	146 (83.9)	
*Diabetes*			
Yes	88 (11.5)	30 (17.2)	*p* = .038
No	679 (88.5)	144 (82.8)	
*Effects of stroke*			
Yes	9 (1.2)	10 (5.8)	*p < .001*
No	760 (98.8)	162 (94.2)	
*Heart disease*			
Yes	41 (5.4)	17 (9.8)	*p* = .027
No	725 (94.6)	156 (90.2)	
*High blood pressure*			
Yes	172 (22.4)	44 (25.7)	*p* = .349
No	596 (77.6)	127 (74.3)	
*Mood disorder*			
Yes	134 (17.5)	61 (31.3)	*p < .001*
No	632 (82.5)	112 (64.7)	
ORAL HEALTH			
*Dental insurance coverage*			
Yes	573 (74.9)	112 (65.1)	*p = .009*
No	192 (25.1)	60 (34.9)	
*Employer insurance*			
Yes	384 (50.4)	56 (32.6)	*p < .001*
No	378 (76.5)	116 (23.5)	
*Non-insured health benefits*			
Yes	140 (18.4)	19 (11)	*p* = .021
No	622 (81.6)	153 (89)	
*Social service insurance*			
Yes	55 (7.2)	35 (20.3)	*p < .001*
No	707 (92.8)	137 (79.7)	
*Regular dental visits*			
Yes	579 (75.8)	97 (56.1)	*p < .001*
No	185 (24.2)	76 (43.9)	
*Dental visit for emergency*			
Yes	109 (14.2)	52 (30.1)	*p < .001*
No	659 (85.8)	121 (69.9)	
*Uncomfortable to eat foods*			
Yes	108 (14)	101 (58)	*p < .001*
No	661 (86)	73 (42)	
*Avoid particular foods*			
Yes	76 (9.9)	89 (51.1)	*p < .001*
No	693 (90.1)	85 (48.9)	
*Persistent pain*			
Yes	86 (11.2)	82 (47.1)	*p < .001*
No	683 (88.8)	92 (52.9)	
*Bleeding gums*			
Yes	190 (24.7)	88 (50.6)	*p < .001*
No	579 (75.3)	86 (49.4)	
*Bad breath*			
Yes	117 (15.2)	72 (41.4)	*p < .001*
No	652 (84.8)	102 (58.6)	
***Lifestyle behaviours***			
*Smoking status*			
Smoker	218 (28.3)	98 (56.3)	*p < .001*
Non-smoker	551 (71.7)	76 (43.7)	
*Alcohol consumption*			
Drinker	623 (81.3)	139 (80.3)	*p* = .765
Non-drinker (last 12 months)	143 (18.7)	34 (19.7)	
***Transportation***			
*Driving last year*			
Yes	634 (82.4)	109 (62.6)	*p < .001*
No	135 (17.6)	65 (37.4)	

*p* values in italic indicate statistical significance

Dental visits were also significantly associated with self-rated oral health. Those who visited dental professionals for check-ups were less likely to report poor self-rated oral health than those who did not (*p* < .001). In contrast, those who made emergency visits were more likely to report poor self-rated oral health than those who did not (*p* < .001). Indigenous who reported poor self-rated oral health were more likely to be uncomfortable eating foods, avoiding particular foods, having persistent pain in their mouth, bleeding gums and bad breath (*p* < .001). Additionally, access to employment based dental coverage was significantly associated with having good self-rated oral health (*p* < .001) while having dental coverage by a social service program was significantly associated with having poorer perceptions of oral health (*p* < .001).

### Predictors of poor oral health in Indigenous adults

As shown in Table 3, being a male, general health as well as regular dental visits, being uncomfortable eating foods, avoiding particular foods, persistent pain, bleeding gums, and being a smoker were all predictive of poor self-rated oral health (− 2 likelihood ratio = 539.75, Nagelkerke R^2^ = 0.47). Indigenous male adults, compared to Indigenous female adults, were 1.75x more likely to report poor self-rated oral health after controlling for age. Indigenous adults with poor general health had a 3.50 (AOR) increased odds of having poor self-rated oral health compared to those with good general health. Additionally, being a smoker resulted a 3.63x increased likelihood of having poor self-rated oral health. With respect to oral health indicators, the adjusted odds of having poor self-rated oral health were 2.39x greater for those who felt uncomfortable eating foods, 3.07x for those avoiding particular foods, 2.46x who reported bleeding gums, (2.46), persistent pain (2.02), and not visiting the dentist regularly (2.02).

**Table 3:** Logistic regression for predicting poor self-rated oral health (N = 943)

Variables	Poor self-rated oral health			
	OR (95% CI)	*p* values	AOR (95% CI)	*p* values
*Sex*				
Male	1.76 (1.11–2.78)	*0.02*	1.75 (1.10–2.78)	*0.02*
Female	1		1	
*Marital Status*				
No Partner	1.08 (0.66–1.76)	0.76	1.04 (0.63–1.71)	0.87
Partner	1		1	
*Education*				
Secondary education or less	1.71 (1.01–2.90)	0.05	1.73 (1.01–2.95)	0.05
Post-secondary education	1		1	
*Income*				
Less than $40,000	0.71 (0.39–1.29)	0.26	0.71 (0.39–1.29)	0.26
$40,000 or more	1		1	
*Employment status*				
Unemployed	1.54 (0.91–2.61)	0.11	1.39 (0.792–2.42)	0.25
Employed	1		1	
*Self-rated general health*				
Poor	3.29 (1.81–5.97)	*< 0.01*	3.50 (1.92–6.38)	*< 0.01*
Good	1		1	
*Self-rated mental health*				
Poor	1.88 (0.95–3.70)	0.07	1.90 (0.96–3.75)	0.06
Good	1		1	
*Anxiety disorder*				
Yes	1.43 (0.78–2.65)	0.24	1.45 (0.79–2.69)	0.23
No	1		1	
*Arthritis*				
Yes	1.03 (0.60–1.74)	0.93	1.03 (0.59–1.81)	0.92
No	1		1	
*Diabetes*				
Yes	0.77 (0.37–1.59)	0.48	0.77 (0.37–1.59)	0.48
No	1		1	
*Effects of stroke*				
Yes	3.26 (0.91–11.65)	0.07	3.38 (0.94–12.17)	0.06
No	1		1	
*Heart disease*				
Yes	0.91 (0.38–2.19)	0.83	0.79 (0.32–1.94)	0.61
No	1		1	
*Mood disorder*				
Yes	0.55 (0.28–1.05)	0.07	0.54 (0.28–1.05)	0.07
No	1		1	
*Dental insurance coverage*				
No	2.33 (0.81–6.73)	0.12	2.50 (0.88–7.15)	0.09
Yes	1		1	
*Employer insurance*				
Yes	0.53 (0.18–1.52)	0.24	0.47 (0.16–1.33)	0.15
No	1		1	
*Non-insured health benefits*				
Yes	1.88 (0.69–5.14)	0.22	1.74 (0.64–4.69)	0.28
No	1		1	
*Social service insurance*				
Yes	0.59 (0.19–1.76)	0.34	0.49 (0.17–1.51)	0.22
No	1		1	
*Regular dental visits*				
No	2.02 (1.16–3.50)	*0.01*	2.02 (1.17–3.49)	*0.01*
Yes	1		1	
*Dental visit for emergency*				
Yes	1.18 (0.66–2.10)	0.58	1.23 (0.67–2.18)	0.49
No	1		1	
*Uncomfortable to eat foods*				
Yes	2.39 (1.28–4.51)	*0.01*	2.39 (1.27–4.52)	*0.01*
No	1		1	
*Avoid particular foods*				
Yes	2.97 (1.53–5.80)	*0.01*	3.07 (1.56–6.03)	*0.01*
No	1		1	
*Persistent pain*				
Yes	1.99 (1.11–3.58)	*0.02*	2.02 (1.12–3.65)	*0.02*
No	1		1	
*Bleeding gums*				
Yes	2.49 (1.54–4.05)	*< 0.01*	2.46 (1.51–4.02)	*< 0.01*
No	1		1	
*Bad breath*				
Yes	1.61 (0.96–2.71)	0.07	1.63 (0.97–2.75)	0.07
No	1		1	
*Smoking status*				
Smoker	3.44 (2.12–5.57)	*< 0.01*	3.63 (2.22–5.93)	*< 0.01*
Non-smoker	1		1	
*Driving last year*				
No	1.34 (0.79–2.32)	0.29	1.29 (0.74–2.26)	0.36
Yes	1		1	

*p* values in italic indicate statistical significance

*OR* odds ratio, *AOR* adjusted odds ratio (adjusted by age), *CI* confidence interval

## Discussion

Almost 12% of the general population reported having poor self-rated oral health which is consistent with prior studies show rates between 7.5 and 15.1%[7, 12, 14]. The findings that Indigenous adults have significantly lower perceptions of their oral health compared to the general population are consistent with prior studies using data from the Canadian Health Measures Survey [12] and prior studies elsewhere [19, 20]. Almost one fifth (18.5%) of Indigenous adults reported having poor self-rated oral health which is lower than reported in prior studies. For example, 28% of Indigenous adults on the Canadian Health Measures Survey [12], 39.9% on the First Nations Oral Health Survey [13], and 34.7% on the Inuit Oral Health Survey [11] reported having poor or fair perceptions of their oral health. It is possible this difference is due to the provision of dental coverage for Indigenous people, especially the Non-Insured Health Benefits Program which is specific to the Indigenous population [21], however, our findings did not show that dental insurance was predictive of poor self-rated oral health, consistent with prior reports [22]. Another possibility is that the number of Indigenous adults in our study who self-rated their oral health as good may be inflated or that they overestimated their oral health. A prior report from the First Nations Oral Health Survey found that 46.8% of Indigenous adults perceived no dental problems although the dentist/examiner reported that 83.1% had poor oral health [13].

Poor self-rated oral health in Indigenous adults were predicted by avoiding certain foods, not being comfortable eating food, bleeding gums, and persistent pain. These findings are consistent with prior studies that found the presence or severe oral health symptoms in Indigenous people, especially pain, are related to poorer oral health perceptions, thus influencing their behaviour to seek dental care [8, 20, 23]. Moreover, this study shows gender differences in self-rated oral health as being an Indigenous male predicted poor self-rated oral health. Better oral health perceptions among females can be attributed to their greater preventative oral health practices (e.g. regular dental visits, more frequent brushing) compared to males [14, 16]. Additionally, our findings show that having poor perceptions of general health predicted having poor self-rated oral health. Our results are consistent with prior studies that show an association between perceptions of general health and oral health [7]. Data suggests that Indigenous people’s oral health is associated with a holistic model of wellness and wellbeing that reflects their cultural values (e.g. balance among physical, mental, emotional, spiritual personal well-being with family, community and natural environment) [23–25]. This is consistent with our findings showing the interconnected nature between wellbeing (general health) with oral health. While there is access to oral health care, it is possible that Indigenous populations do not identify with Western practices of medicine. Instead, to improve the likelihood that Indigenous people will seek care, oral health providers should incorporate a holistic model of wellness that reflect the values and traditions of Indigenous people. Prior research shows that culturally adapted community-based oral health interventions (including care being provided by Indigenous care providers) enhance the success of preventative dental programs, reduce dental caries and periodontal diseases, as well as improve oral health literacy [23, 26].

Being a smoker predicted poor oral health perceptions. In fact, more than 30% smoked and this likely increases the risk of oral health issues related to periodontal diseases [27] and oropharyngeal cancer [28]. The negative effects of smoking on oral health has been well documented with multiple studies showing that smoking is associated with chronic periodontitis [27] and oral-facial pain [29]. Furthermore, smoking has been also associated with several general health problems, such as COPD, coronary heart disease, and cancer [28, 30, 31]. Given that smoking was associated with poor self-rated oral health, as well as with general health, it is possible that smoking cessation might improve general wellbeing, as well as oral health given the strong association between general and oral health. Indigenous adults with poor self-rated oral health were also more likely to make emergency visits to the dentist and less likely to seek dental care for check-ups. If Indigenous adults would have made more visits to the dentist to address oral health problems and to reduce the progression of oral health issues, it is likely the number of oral health issues reported in this study, as well as emergency visits, could be reduced. This is consistent with prior observational studies in the general population that show regular dental visits result in better clinical and self-rated oral health [32, 33]. However, visiting the dentist may have been difficult as about 40% of those having poor self-rated oral health did not have access to a vehicle, and reported anxiety disorders, such as phobia or panic, which can influence their willingness to seek care. Additionally, there are socioeconomic issues related to employment and income. Our study shows that reporting good self-rated oral and general health were commonly found among those with higher incomes and employment. Being employed with a stable and good income, may enable healthier lifestyle choices and ultimately result in greater self-rated general health, as well as oral health [6, 34], and may lead to more frequent preventative visits to the dentist rather than for emergency care.

There are a few limitations to the study. While the CCHS is considered a representative sample, we are unsure how many community dwelling Indigenous adults chose not to complete the survey. Additionally, this survey was not completed by anyone living on-reserve where oral health issues have been reported to be worse [25]. Additionally, the CCHS did not collect data on the number of decayed/missing/filled teeth which has been associated with poor perceptions of oral health in prior studies [35]. Moreover, there is also a risk of recall and social desirability bias when participants complete surveys. And lastly, the CCHS is a cross-sectional survey precluding the ability to determine causality.

Future studies should attempt to develop interventions/modules to help improve the understanding and education surrounding oral health in Indigenous populations given the strong association we found between income, general health and oral health care practices. As these factors are likely all intertwined, identifying ways to encourage Indigenous adults to visit the dentist for regular appointments rather than emergency visits would help improve their overall oral health, and perhaps their general health. Additionally, further exploration of the oral health practices of Indigenous adults living on-reserve communities is warranted given the limited data available.

## Conclusions

Findings from this study show that a myriad of factors are related to oral health perceptions in Indigenous adults. Interventions are needed to ensure Indigenous adults can access oral health care services on a regular basis to improve oral health care outcomes in this population.

## Data Availability

The dataset pertaining to the CCHS is publicly available and can be provided from the corresponding author on request. The dataset can be assessed using the following link: http://odesi2.scholarsportal.info/webview/.

## Abbreviations

CCHS: Canadian Community Health Survey
OR: Odds ratio
AOR: Adjusted odds ratio

## References

[1] Hescot P (2017). The new definition of oral health and relationship between oral health and quality of life. Chin J Dent Res.

[2] Dörfer C, Benz C, Aida J, Campard G (2017). The relationship of oral health with general health and NCDs: a brief review. Int Dent J.

[3] Spanemberg JC, Cardoso JA, Slob, Lo J, Slob EMGB, López-López J (2019). Quality of life related to oral health and its impact in adults. J Stomatol Oral Maxillofac Surg.

[4] World Health Organization. Oral health. 2020. https://www.who.int/news-room/fact-sheets/detail/oral-health.

[5] Kakudate N, Morita M, Fukuhara S, Sugai M, Nagayama M, Kawanami M (2010). Application of self-efficacy theory in dental clinical practice. Oral Dis.

[6] Locker D (2009). Self-esteem and socioeconomic disparities in self-perceived oral health. J Public Health Dent.

[7] Bassim CW, MacEntee MI, Nazmul S, Bedard C, Liu S, Ma J (2020). Self-reported oral health at baseline of the Canadian Longitudinal Study on aging. Community Dent Oral Epidemiol.

[8] Mauricio HA, Moreira RS (2020). Self-perception of oral health by indigenous people: an analysis of latent classes. Cienc e Saude Coletiva.

[9] Hau KPH, Currie BL, Ng SPY, Le N, Poh CFY (2017). Oral health status and possible explanatory factors of an inner-city low-income community. J Dent Sci.

[10] Jessani A, Laronde D, Mathu-muju K, Brondani M. Self-perceived oral health and use of dental services by pregnant women in Surrey, British Columbia. 2016. p. 1–11. 28240578

[11] Health Canada, Nunavut Tunngavik Incorporated, Nunatsiavut Government, Inuvialuit Regional Corporation ITK. Inuit Oral Health Survey report 2008–2009. 2011. https://www.tunngavik.com/files/2011/05/inuitoralhealthsurveyreport_2008-09.pdf.

[12] Health Canada. Summary report on the findings of the oral health component of the Canadian Health Measures Survey 2007–2009. 2010. http://www.caphd.ca/sites/default/files/CHMS-E-summ.pdf.

[13] The First Nations Information Governance Centre. Report on the Findings of the First Nations Oral Health Survey (FNOHS) 2009–2010. 2012. https://fnigc.ca/sites/default/files/docs/fn_oral_health_survey_national_report_2010.pdf.

[14] Zangiabadi S, Costanian C, Tamim H (2017). Dental care use in Ontario: The Canadian community health survey (CCHS). BMC Oral Health.

[15] Blanchard AK, Wang X, El-gabalawy H, Tan Q, Orr P, Elias B (2012). Oral health in a First Nations and a non-Aboriginal population in Manitoba. Int J Circumpolar Health.

[16] Mehra VM, Ali-Hassan Y, Tamim H, Costanian C (2020). Prevalence and factors associated with visiting the dentist only for emergency care among Indigenous people in Ontario. J Immigr Minor Health.

[17] Statistics Canada. Community Health Survey-Annual Component (CCHS). 2020. https://www23.statcan.gc.ca/imdb/p2SV.pl?Function=getSurvey&SDDS=3226.

[18] Government of Canada. What is Indian status. Government of Canada. 2020. https://www.sac-isc.gc.ca/eng/1100100032463/1572459644986. Accessed 21 May 2021.

[19] Schuch HS, Haag DG, Kapellas K, Arantes R, Peres MA, Thomson WM (2017). The magnitude of Indigenous and non-Indigenous oral health inequalities in Brazil, New Zealand and Australia. Community Dent Oral Epidemiol.

[20] Tynan A, Walker D, Tucker T, Fisher B, Fisher T (2020). Factors influencing the perceived importance of oral health within a rural Aboriginal and Torres Strait Islander community in Australia. BMC Public Health.

[21] Zivkovic N, Aldossri M, Gomaa N, Farmer JW, Singhal S, Quiñonez C (2020). Providing dental insurance can positively impact oral health outcomes in Ontario. BMC Health Serv Res.

[22] Office of the Auditor General of Canada. Report 4-Oral Health Programs for First Nations and Inuit-Health Canada. 2017. https://www.oag-bvg.gc.ca/internet/English/parl_oag_201711_04_e_42669.html#p30. Accessed 6 Dec 2020.

[23] Mathu-muju KR, Mcleod J, Donnelly L, Harrison R, Macentee MI, Mcleod J (2017). The perceptions of first nation participants in a community oral health initiative. Int J Circumpolar Health.

[24] Martin D, Mcnally M, Castleden H, Clarke M, Wall D, Ley M (2018). Linking Inuit knowledge and public health for improved child and youth oral health in NunatuKavut. JDR Clin Transl Res.

[25] Lawrence HP, Cidro J, Isaac-Mann S, Peressini S, Maar M, Schroth RJ (2016). Racism and oral health outcomes among pregnant Canadian aboriginal women. J Health Care Poor Underserved.

[26] Tiwari T, Jamieson L, Broughton J, Lawrence H, Batliner T, Arantes R (2018). Reducing Indigenous oral health inequalities: a review from 5 nations. J Dent Res.

[27] Wickholm S, Rosaria Galanti M, Söder B, Gilljam H (2003). Cigarette smoking, snuff use and alcohol drinking: coexisting risk behaviours for oral health in young males. Community Dent Oral Epidemiol.

[28] Harris RE (2019). Epidemiology of chronic disease: global perspectives.

[29] Millar WJ, Locker D. Smoking and oral health status. J Can Dent Assoc (Tor). 2007;73. https://pubmed.ncbi.nlm.nih.gov/17355806/. Accessed 18 Feb 2021. 17355806

[30] Plurphanswat N, Kaestner R, Rodu B (2017). The effect of smoking on mental health. Am J Health Behav.

[31] Taylor G, McNeill A, Girling A, Farley A, Lindson-Hawley N, Aveyard P (2014). Change in mental health after smoking cessation: systematic review and meta-analysis. BMJ.

[32] Aldossary A, Harrison VE, Bernabé E (2015). Long-term patterns of dental attendance and caries experience among British adults: a retrospective analysis. Eur J Oral Sci.

[33] Åstrøm AN, Ekback G, Ordell S, Nasir E (2014). Long-term routine dental attendance: influence on tooth loss and oral health-related quality of life in Swedish older adults. Community Dent Oral Epidemiol.

[34] Farmer J, Phillips RC, Singhal S, Quiñonez C (2017). Inequalities in oral health: understanding the contributions of education and income. Can J Public Health.

[35] Thomson WM, Mejia GC, Broadbent JM, Poulton R (2012). Construct validity of locker’s global oral health item. J Dent Res.

